# FRD-CNN: Object detection based on small-scale convolutional neural networks and feature reuse

**DOI:** 10.1038/s41598-019-52580-0

**Published:** 2019-11-08

**Authors:** Wei Li, Kai Liu, Lin Yan, Fei Cheng, YunQiu Lv, LiZhe Zhang

**Affiliations:** School of Computer Science and Technology, Xidian University, Xi’an, 710071 China

**Keywords:** Information technology, Computer science

## Abstract

Most of the recent successful object detection methods have been based on convolutional neural networks (CNNs). From previous studies, we learned that many feature reuse methods improve the network performance, but they increase the number of parameters. DenseNet uses thin layers that have fewer channels to alleviate the increase in parameters. This motivated us to find other methods for solving the increase in model size problems introduced by feature reuse methods. In this work, we employ different feature reuse methods on fire units and mobile units. We solved the problem and constructed two novel neural networks, fire-FRD-CNN and mobile-FRD-CNN. We conducted experiments with the proposed neural networks on KITTI and PASCAL VOC datasets.

## Introduction

Object detection is a basic task of image processing. Early object detection algorithms mainly relied on handcrafted features and shallow machine learning algorithms. These methods were vulnerable to overfitting and often included a large number of calculations. Recently, convolutional neural networks (CNNs) have been used to learn feature representations from images automatically, and this has been the dominant approach for object detection. In this paper, two novel CNNs are proposed for object detection.

Different layers in a CNN contain feature information of different dimensions. Lower layers contain more positional information but relatively less high-dimensional semantic information. Higher layers contain more high-dimensional semantic information but relatively less positional information. It is important to properly make use of all the feature map information of different layers to achieve high detection precision.

**Figure 1:**
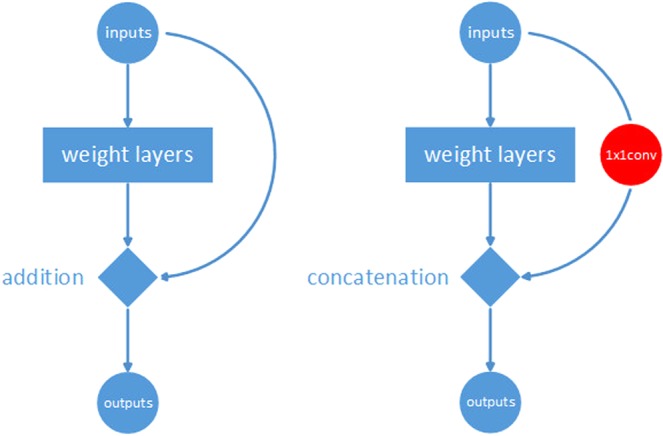
Basic feature reuse methods. Left: bypass; right: concatenation.

Usually, bypass or concatenation paths are added to the network. In fact, both bypass and concatenation connectivity are essentially types of feature reuse approaches, each with different good properties. In addition to feature reuse, bypass paths have been proven to be able to overcome the vanishing gradient problem^1^. Because the vanishing gradient problem occurs at deeper layers of a network, it is suitable to place bypass layers at the bottom of a CNN. Because concatenation paths can best preserve the feature information of different layers used for detection, it is preferable to place concatenation layers at the top or near the detection modules of a network. The two basic feature reuse methods are shown in Fig. 1.

Dense connectivity is a method for improving the performance of a neural network, but it increases the number of parameters. DenseNet^2^ uses thin layers that have fewer channels to alleviate the increase in parameters. This motivated us to find other methods for solving the increase in model size problems introduced by dense connectivity. We analyzed the model increase by dense connectivity. We found that applying dense connectivity to fire units and mobile units led to a small parameter increase. They are more suitable for dense connectivity. Formally, assume the shape of input tensors of layer *i* are *w* * *h* * *c* and *w*′ * *h*′ * *c*′, where *w* * *h* * *c* is the shape of tensor from layer *i* − 1, and *w*′ * *h*′ * *c*′ is the shape of concatenated tensors from the layers before layer *i* − 1. for the normal 3 * 3 convolution layer, the increased parameters are *c*′ * (3 * 3) * *c* + *c*. For fire units, the increased parameters are *c*′ * (1 * 1) * *c*″ + *c*″, where *c*″ is the number of channels of the current 1 * 1 convolution layer, and it is typically less than *c*. Therefore, the increase in parameters is less than 9× that of normal 3 * 3 convolution units. For mobile units, the increased parameters are *c*′ * (3 * 3) * 1 + *c*′. Therefore, the increased parameters are approximately (*c*″ − 1)× less than those of normal 3 * 3 convolution units. As a result, it is suitable to use dense connectivity to improve the performance of models built on fire units and mobile units.

In our work, a method leveraging the advantages of feature reuse methods is proposed. First, CNNs based on “fire” or “mobile” units are constructed. They are used as the backbone network of the whole object detection network. Next, we investigate the two feature reuse methods in depth on this small-scale CNN to best exploit the feature information of each layer. Finally, a detection network is constructed based on the backbone network. We tested our proposed network on the KITTI^3^ and PASCAL VOC^4^ datasets.

The main contributions of this paper are as follows:

First, to facilitate the construction of our final detection network, two backbone CNNs named fire-unit-based feature reuse CNNs (fire-FR-CNNs) and mobile-unit-based feature reuse CNNs (mobile-FR-CNNs) are proposed for feature extraction.

Second, the feature reuse method is investigated in depth. A feature reuse method that leverages both bypass and “complete” concatenation paths is proposed.

Third, based on the above two works, two feature reuse detection CNNs (FRD-CNNs) used for object detection are proposed. We conducted experiments on KITTI and PASCAL VOC datasets with the proposed neural networks, which show that the proposed methods achieved better performance compared to baseline models.

## Related Work

Cai *et al*. proposed a multiscale CNN (MS-CNN)^5^. They proposed that the scale of an object in an image varies. Previous methods always implemented object detection only at the end of a CNN. This resulted in missed detection of objects if they were too small in size. Because the pixels on feature maps of lower layers have a small receptive field, MS-CNN is more suitable for small object detection; however, the pixels on feature maps of higher layers have a large receptive field, which is more suitable for large object detection. The authors designed a CNN that implemented the detection task at different layers of the network. The final detection result was the combination of the detection results of the different layers. This method is more resistant to frequent scale changes in object detection tasks. In the present paper, we show that through complete concatenation, implementing the detection task only at the end of the CNN has an effect similar to that seen in the MS-CNN.

Yang *et al*. proposed scale-dependent pooling and layerwise cascaded rejection classifiers^6^. By selecting pooling regions for candidate object proposals with proper scale, scale-dependent pooling can improve detection accuracy. Cascaded rejection classifiers exclude “easy” negative object proposals in a cascaded manner, thus improving detection accuracy. While this method obtained relatively high detection precision, it increased the number of calculations.

Ren *et al*. proposed recurrent rolling convolutions (RRCs)^7^. The authors proposed that feature maps in lower layers had higher resolution and more positional information. Furthermore, feature maps in higher layers have more high-dimensional semantic information. Previous works have often implemented detection tasks using feature information from the last layer. In this work, feature information in each layer was fused in a recurrent rolling manner, which greatly improved detection performance. While this model achieved top detection accuracy on the KITTI dataset, it needed to run for several epochs to fuse the feature information of each layer with that of other layers.

Feature pyramid networks (FPNs)^8^ fuse high-level semantic information to low-level positional information. FPN and its derived neural networks have the advantages of detecting small objects and objects with large-scale variations. Kong *et al*. proposed deep feature pyramid reconfiguration for object detection^9^, which consists of global attention and local reconfigurations methods. Both global attention and local reconfigurations are lightweight, so the proposed model achieved consistent and significant improvements without losing real-time processing speed. Sun *et al*. proposed feature pyramid reconfiguration with consistent loss for object detection^10^. They reshaped the standard cross-entropy loss and designed a novel consistent loss (CL). It achieved more accurate object localization. Pang *et al*. proposed the efficient featurized image pyramid network for single-shot detector^11^, in which a lightweight featurized image pyramid network was introduced to construct a multiscale feature representation. In addition, StairNet^12^ and two variants of a context-aware single-shot detector^13^ were proposed to improve the detection performance of small objects.

Han *et al*. proposed network pruning^14^. In this work, a CNN was trained, and parameters in the model below a predefined threshold were set to zero to construct a sparse model. Then, the sparse model was trained for a few iterations. Finally, a smaller model was produced.

Hinton *et al*. proposed knowledge distillation^15^. The authors trained a small student CNN beginning with training a large teacher CNN. The small network was trained to mimic its teacher network. The loss function of the student network was the difference between the outputs of the student network and the teacher network, thus a “soft” target with respect to their original loss function. This yielded better results than directly training the student CNN through the original “hard” target.

Howard *et al*. proposed a streamlined network named MobileNet^16^. It used a depthwise separable convolution, which is a combination of separable convolution and pointwise convolution. These two steps are in essence a filtering stage (separable convolution) and a combination stage (pointwise convolution). Based on this strategy, a small-scale network was constructed. Later, ShuffleNet^17^ was proposed. In ShuffleNet, a channel shuffle operation is imposed after each pointwise convolution to force feature map information exchange between different channels to improve detection performance.

Iandola *et al*. proposed a small network named SqueezeNet^18^. SqueezeNet was designed to be especially small based on three strategies. First, 3 × 3 filters were replaced with 1 × 1 filters. Second, the number of input channels was decreased to 3 × 3 filters. Last, downsampling occurred late in the network so that convolution layers had large activation maps. Based on these three strategies, a “fire” module was constructed. The fire module from which SqueezeNet was constructed was comprised of a squeeze layer and an expand layer. By using the fire units as the basic module of SqueezeNet, the number of parameters of SqueezeNet were largely decreased.

Based on SqueezeNet, a network used for object detection named SqueezeDet^19^ was proposed. It used SqueezeNet as its backbone network for feature extraction, and a fully convolutional detection network was designed for final object detection. This model can be trained end-to-end and is small in size. Its detection speed is especially high.

Densely connected convolutional networks (DenseNet) were proposed in^2^. In this work, each layer was connected to all other layers in a feed-forward fashion, which yielded a densely connected network. DenseNet alleviates the vanishing gradient problem, strengthens feature propagation, and encourages feature reuse.

## Methods

This section addresses the backbone network design, feature reuse methods analysis, and final detection network design.

### Backbone network fire-FR-CNN design

The fire-FR-CNN is built upon fire units. Specifically, in our work, they should obey some prerequisites to incorporate bypass and complete concatenation paths into the model.

#### Fire unit

A fire unit is comprised of a squeeze layer as input and two parallel expand layers as output. The squeeze layer is essentially a set of 1 × 1 convolution filters that takes a tensor with a large channel size as input and outputs a tensor with a small channel size while keeping the shape and batch of the tensor unchanged. The expand layer is the concatenation of a 1 × 1 and 3 × 3 convolution layer that takes the compressed tensor from the squeeze layer as input and outputs a tensor with a large channel size. The expand layer is used to retrieve the rich feature information. Let the number of filters of the squeeze layer be less than the number of filters of the two expand layers, and by alternating the use of the squeeze layers and expand layers, the number of parameters can be effectively reduced without losing too much accuracy.

#### Mobile unit

The standard convolution layer has the effect of extracting features through convolution kernels and rearranging the features to produce a new feature representation. Mobile units separate standard convolutions into two parts: a depthwise separable convolution layer and a 1 × 1 convolution layer. The depthwise separable convolution layer applies a single filter to each input channel. The pointwise convolution acts as a combine operation on the outputs of the depthwise convolution layer. Compared to standard 3 × 3 convolution layers, mobile units largely decrease both computations and number of parameters.

#### Fire-FR-CNN layers

To incorporate bypass and complete concatenation paths into the model, we divide the layers into four blocks. Let fire2 and fire3 belong to block1; fire4 and fire5 belong to block2; fire6 to fire9 belong to block3; and fire10 to fire13 belong to block4. Let the output dimensions of fire modules in block1 be the same. Thus, all feature maps in this block can be added to other feature maps. This design principle is also applied to block2. Block3 is designed without pooling layers so that all feature maps in this block can be concatenated to other feature maps. This design method is also applied to block4.

#### Mobile-FR-CNN layers

We apply both bypass and complete concatenation paths to construct mobile-FR-CNN layers. The bypass and complete concatenation connectivity and network parameters are shown in Table 1.

**Table 1:** Mobile-FR-CNN parameters used for each layer.

Type/Stride	Filter shape	Number of parameters
Conv/s2	3 × 3 × 32	896
Conv dw/s1	3 × 3 × 32 dw	320
Conv/s1	1 × 1 × 64	2,112
Conv dw/s2 (skip connection)	3 × 3 × 64 dw	640
Conv/s1	1 × 1 × 128	8,320
Conv dw/s1	3 × 3 × 128 dw	1,280
Conv/s1	1 × 1 × 128	16,512
Conv dw/s2 (skip connection)	3 × 3 × 128 dw	1,280
Conv/s1	1 × 1 × 256	33,024
Conv dw/s1	3 × 3 × 256 dw	2,560
Conv/s1	1 × 1 × 256	65,792
Conv dw/s2	3 × 3 × 256 dw	2,560
Conv/s1	1 × 1 × 512	131,584
Conv dw/s2	3 × 3 × 512 dw	5,120
Conv/s1	1 × 1 × 512	262,656
Conv dw/s1 (concat connection)	3 × 3× * dw	2,406,912
Conv/s1	1 × 1 × 512	
Conv dw/s1	3 × 3 × 512 dw	5,120
Conv/s1	1 × 1 × 512	262,656
—	—	3.2M (total)

### Feature reuse methods analysis

There are two basic feature reuse methods: bypassing and concatenation. Bypassing has been proven to be able to overcome the vanishing gradient problem. Because the vanishing gradient problem occurs at deep layers of a network, it is suitable to place bypass layers at the deep (beginning) modules of the network. Additionally, because concatenation can best preserve the feature information of different layers used for detection, it is preferable to place concatenation layers at the upper layers or near the detection modules of a network. Based on this reasoning, a network containing four blocks is proposed. The first two blocks are designed considering bypass paths, while each fire unit in the last two blocks is completely concatenated with other fire modules in the same block.

#### Bypass connections

Bypass connections take the output of the (*l* − 1)^*th*^ layer as the input of the *l*^*th*^ fire module, which can be formulated as *x*_*l*_ = *S*(*x*_*l*−1_). Feature reuse through bypass connections can be formulated as follows:

1 $$\begin{array}{l}{x}_{l}=S({x}_{l-1})+{x}_{l-1}.\end{array}$$

*S* is a nonlinear function representing the transformation in a fire module.

**Figure 2:**
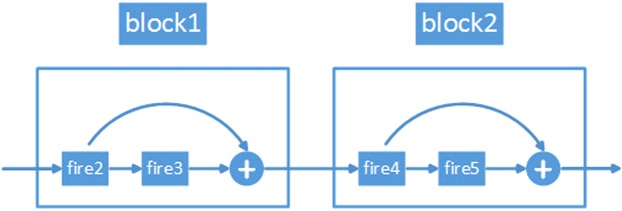
Design of block1 and block2.

In block1, the output of fire2 is added to the output of fire3 as the input of fire4 in block2. In block2, the output of fire4 is added to the output of fire5 as the input of the succeeding layer. This structure is shown in Fig. 2.

#### Complete concatenation

To further improve the information flow and best utilize feature map information produced by each fire block. Inspired by DenseNet, we introduce complete concatenation, where each output of a fire module is concatenated to all subsequent “fire” modules within the same block.

**Figure 3:**
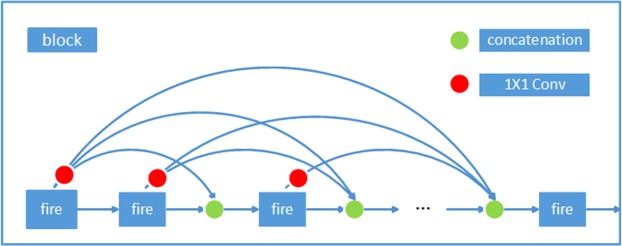
Design of block3 (1).

Directly concatenating a fire module to all following fire modules in the same block will lead to a rapid increase in the number of channels passed into a fire module, and the model will become unstable given a long sequence of fire modules. This problem can be resolved by adding a 1 × 1 convolutional layer before each concatenation to confine the module size if necessary; however, this can lead to a slight decrease in detection precision (Fig. 3).

The *l*^*th*^ fire module receives the feature maps of all preceding fire modules, *x*_0_, *x*_1_, …, *x*_*l*−1_, as input:

2 $$\begin{array}{l}{x}_{l}=S([C({x}_{0}),C({x}_{1}),\ldots ,C({x}_{l-2}),{x}_{l-1}]),\end{array}$$

where *C* refers to a 1 × 1 convolution. Figure 3 illustrates the layout of this method schematically.

Another method for solving this problem is to confine the concatenation within several small blocks. By connecting these blocks in a cascade manner, a CNN can also be built. The *l*^*th*^ fire module receives the feature maps of all preceding fire modules in the same block, *x*_0_, *x*_1_, …, *x*_*l*−1_, as input:

3 $$\begin{array}{l}{x}_{l}=S({x}_{0},{x}_{1},\ldots ,{x}_{l-2},{x}_{l-1}).\end{array}$$

**Figure 4:**
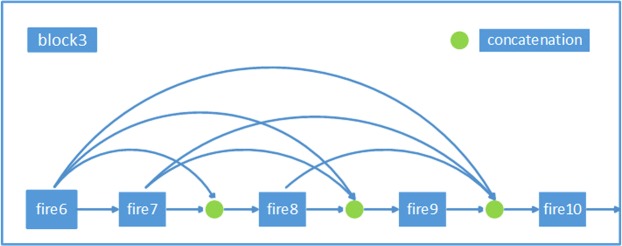
Design of block3 (2).

Figure 4 illustrates the layout of the resulting complete concatenation.

**Figure 5:**
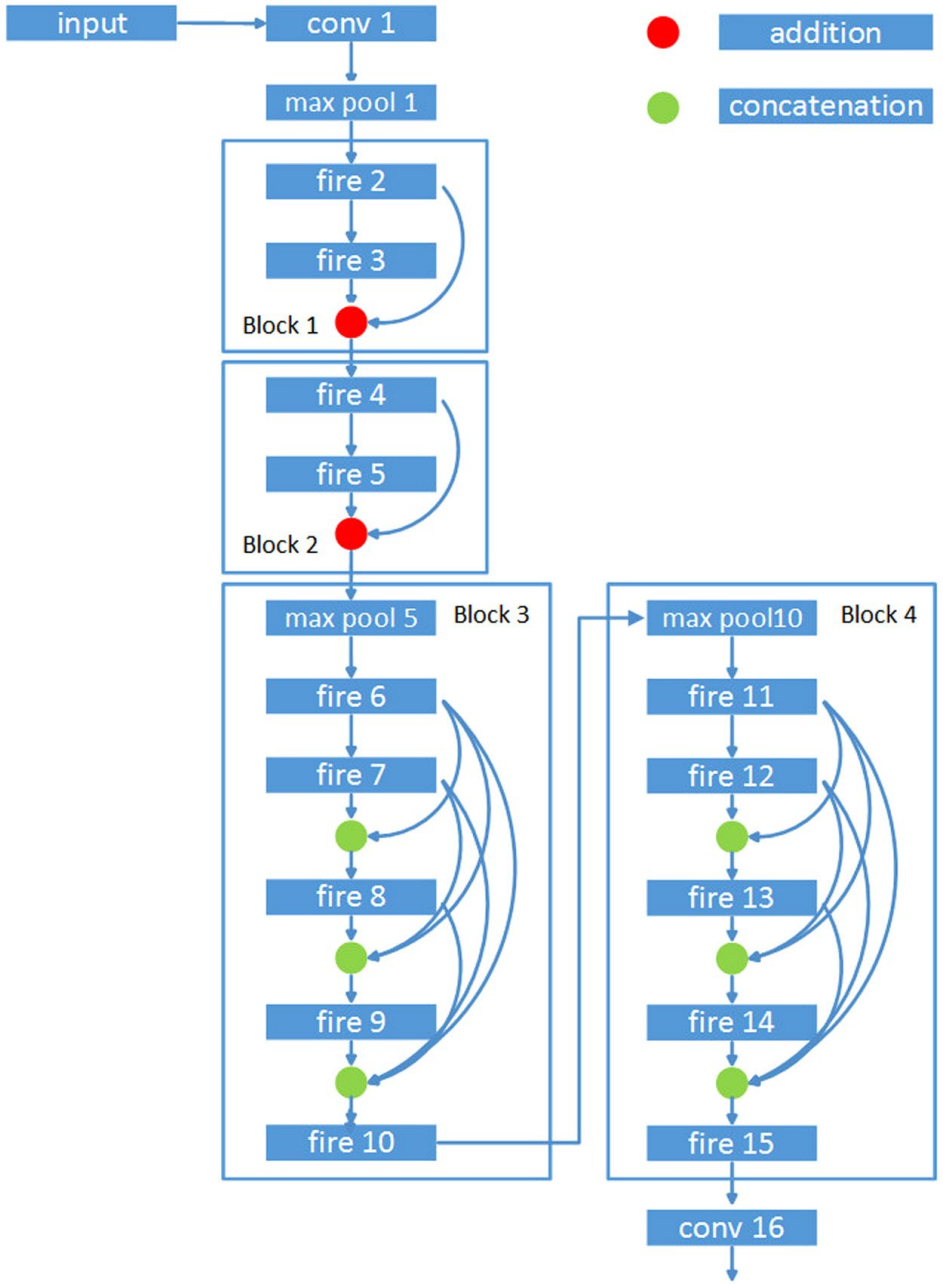
Flow chart of the fire-FR-CNN.

We now describe the fire-FR-CNN architecture. As illustrated in Fig. 5, the fire-FR-CNN starts with a single convolutional layer (conv1), followed by 14 fire modules (fire2–15), ending with a final convolutional layer (conv16). The number of kernels in each fire module gradually increases from fire2 to fire15. Max-pooling with stride 2 is placed after conv1, fire5, and fire10; placing each pooling layer at relatively late layers of the network is based on Strategy 3 mentioned in Section 2.2. The detailed parameters of fire-FR-CNN are introduced in the following paragraphs.

Number of kernels: In common CNN design, the number of kernels in the lower layers is relatively small. The number of kernels increases slowly to extract high dimensional semantic information. We follow this design principle and start with only 96 kernels. We slowly increase the number of kernels. In fire modules, the number of kernels of the convolution layer in the squeeze layer is much smaller than the summation of the number of kernels of the two convolution layers in the expand layer. This method follows the design principle of fire units to construct a relatively small and compact framework; we set the number of kernels of conv16 to 9 × (*c* + 1 + 4) to obtain an output vector of proper shape for both classification and location.

Kernel size: There are three types of kernels with different kernel sizes used for different objectives. First, in conv1, the kernel size is 7 × 7. It is a kernel with a relatively large size. This is chosen because the size of the object feature of the first layer is very large, and we need a large kernel to cover a relatively large receptive field. Second, as a generic design principal, 3 × 3 kernels are used to extract high-dimensional semantic information. We followed this design principle when designing conv16 and each convolution layer in the expand layer. Third, 1 × 1 kernels are used in both squeeze layers and expand layers. In squeeze layers, the number of 1 × 1 kernels is small, so it can extract more useful information and discard redundant information. In expand layers, the 1 × 1 kernels can enhance feature fusion in different channels and improve the nonlinear representation ability of a network^20^.

Fire-FR-CNN initialization: The design of modern deep neural networks relies largely on transfer learning. It makes the training fast convergence and prevents the training from overfitting. Because the backbone network is used to extract feature information of different dimensions, feature information of lower dimensions are very similar, and feature information of higher layers becomes very different. Therefore, we only use pretrained models to initialize the first few layers of a new network. We already have a pretrained SqueezeNet+ model from the previous work on SqueezeDet. We make use of this pretrained model when training the proposed model. Fire-FR-CNN is initialized with pretrained SqueezeDet+ from the first convolution layer to fire 7. The layers from fire 8 to the last convolution layer are initialized with random values.

Addition/concatenation: Addition and concatenation paths can fuse and strengthen information flow from different layers. This has been confirmed by previous works, such as those involving ResNet^1^ and DenseNet^2^. In this work, we take the advantages of both addition and concatenation paths. The add operation does not affect the number of channels within the next output layer, but the concatenation operation does not have this property. If we use the concatenation operation within these layers, we cannot take advantage of the pretrained model. Therefore, the addition is imposed on the lower layers of the framework. For a pretrained model, we use only its lower layers to initialize the object detection framework. Thus, the upper layers of the framework are initialized by random numbers, and we utilize concatenation to make use of information from different layers. Through this strategy, the framework makes use of both the pretrained model and add/concatenation to improve performance. We conducted experiments on different add/concatenation settings, such as using only concatenation and randomly initializing the network. We found that the mAP decreased, and the training took longer to converge.

### Final detection network (fire-FRD-CNN) design

#### Detection pipeline

Because a single-stage detection pipeline is fast in detection, we adopt this type of detection pipeline. Inspired by SSD^21^, the detection network is composed of fully convolutional layers. This type of detection network is small in size and fast in detection, but we implement the detection network only at the last layer of the model. Although the detector is implemented only in the last layer, because feature map information from the lower layers of the model are concatenated to higher layers of the model, we can process information directly from lower layers. This is similar to detectors that implement the detection task at multiple layers (such as in SSD). Because there is only one detector used in our work, this leads to a relatively small module size and faster detection speed.

**Figure 6:**
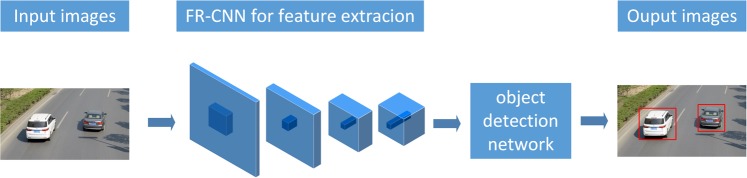
Flow chart of the fire-FRD-CNN.

As shown in Fig. 6, the FRD-CNN takes an image as input, and the fire-FR-CNN is responsible for feature extraction from the image. Then, the feature map is passed to the object detection network to generate the final object detection result.

#### Object detection network

**Figure 7:**
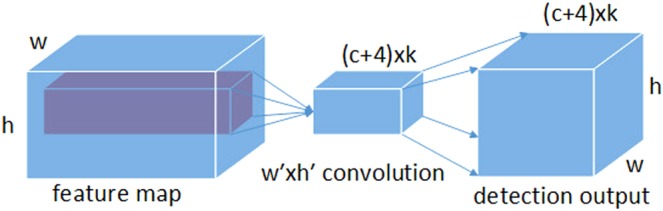
Object detection module design.

As shown in Fig. 7, in the object detection network, bounding boxes around each *w* × *h* pixel on the feature map are generated, and confident scores associated with these bounding boxes are calculated. We refer to these bounding boxes as anchors. Specifically, for a bounding box labeled as k, *c* class scores and four offsets with respect to the anchors are calculated. This produces a total of (*c* + 1 + 4)*k* kernels applied to each position in the feature map. Through a proper padding/striding strategy, the output of the object detection network keeps the same spatial dimensions as the feature map and leads to (*c* + 1 + 4)*kwh* outputs for a *w* × *h* feature map.

Each pixel on a feature map is associated with a rectangle center on the input image. Thus, we describe each anchor with four scalars as $({\hat{x}}_{i},{\hat{y}}_{j},{\hat{w}}_{k},{\hat{h}}_{k})$, where $\hat{x}$, $\hat{y}$ are coordinates of the center of the anchor. The coordinate of the predicted bounding box can be calculated using the equation below:

4 $$\begin{array}{rcl}{x}_{i} & = & {\hat{x}}_{i}+{\hat{w}}_{k}{\Delta }_{{x}_{ijk}},\\ {y}_{j} & = & {\hat{y}}_{j}+{\hat{h}}_{k}{\Delta }_{{y}_{ijk}},\\ {w}_{i} & = & {\hat{w}}_{k}\,\exp \,({\Delta }_{{w}_{ijk}}),\\ {h}_{i} & = & {\hat{h}}_{k}\,\exp \,({\Delta }_{{h}_{ijk}}),\end{array}$$

where Δ is the relative coordinates on anchors for transforming the anchors into a predicted bounding box. Let the size of the input feature map fed into the object detection network be *S*(*w*, *h*, *f*), where *w*, *h*, and *f* are the width, height, and number of channels, respectively, of the input feature map. Assume that the kernels of the object detection network are of size *S*(*w*′, *h*′), where *w*′ and *h*′ are the width and height of the kernel, respectively. Thus, the parameters required by the object detection network are *fwhK*(*c* + 1 + 4). Compared with a detection network implemented by fully convolutional layers that need *whf* × *ch* parameters, where *ch* refers to the number of neurons in a fully connected layer, there are relatively few parameters needed. This also leads to a small fire-FRD-CNN model size.

Similar to SSD, our fire-FRD-CNN can be trained end-to-end. To train the fire-FRD-CNN for detection, a multitask loss function is defined:

5 $$\begin{array}{l}L(x,c,\Delta ,{\Delta }^{g})=\frac{1}{N}({L}_{conf}(x,c)+{L}_{loc}(x,\Delta ,{\Delta }^{g})).\end{array}$$

The first term of the loss function is confidence loss over multiple class confidences. The second term of the loss function is bounding box regression.

### Accession codes

(https://github.com/liweiac/fire-FRD-CNN).

## Results

### Experimental setup

The parameters used for each layer are listed in Table 2. For conciseness, some of the information about the network is provided in the following paragraph.

1. Padding strategy. Conv1, maxpool1, maxpool5, and maxpool10 are not padded. Conv16 and all layers, including squeeze layers and expanding layers in the fire modules, are padded to output a feature layer that has the same size as the input feature layer.
2. In conv16, the number of kernels is 9 × (*c* + 1 + 4), where *c* refers to the number of classes in the dataset.
3. s_1×1_ refers to the number of kernels of the convolution layer in the squeeze layers. e_1×1_ and e_3×3_ refer to the number of kernels of two parallel convolution layers in the expand layers.

**Table 2:** Fire-FR-CNN parameters used for each layer.

Layer	Output dimension	Kernel size	Kernel number	s_1×1_ number	e_1×1_ number	e_3×3_ number	Stride	Number of parameters
conv1	618 × 185	7 × 7	96	—	—	—	2	14,208
maxpool1	308 × 92	3 × 3	—	—	—	—	2	—
fire2	308 × 92	—	—	96	64	64	—	70,880
fire3	308 × 92	—	—	96	64	64	—	73,952
fire4	308 × 92	—	—	192	128	128	—	270,784
fire5	308 × 92	—	—	192	128	128	—	295,360
maxpool5	153 × 45	3 × 3	—	—	—	—	2	—
fire6	153 × 45	—	—	288	192	192	—	627,360
fire7	153 × 45	—	—	288	192	192	—	664,224
fire8	153 × 45	—	—	288	192	192	—	774,816
fire9	153 × 45	—	—	288	192	192	—	885,408
fire10	153 × 45	—	—	384	256	256	—	1,573,760
maxpool10	76 × 22	3 × 3	—	—	—	—	2	—
fire11	76 × 22	—	—	384	256	256	—	1,180,544
fire12	76 × 22	—	—	384	256	256	—	1,180,544
fire13	76 × 22	—	—	384	256	256	—	1,377,152
fire14	76 × 22	—	—	384	256	256	—	1,573,760
fire15	76 × 22	—	—	512	384	384	—	3,015,936
conv16	76 × 22	3 × 3	9 × (*c* + 1 + 4)	—	—	—	1	497,736
—	—	—	—	—	—	—	—	14M (total)

### Experiments on the KITTI dataset

The performance of our proposed fire-FRD-CNN was evaluated on the KITTI dataset^4^. KITTI contains three types of objects: cars, pedestrians, and cyclists. Each object is also classified by its difficulty to detect: easy, moderate, and hard. We tested the fire-FRD-CNN on all of these data categories. We analyzed the fire-FRD-CNN by mean average precision (mAP), recall, and detection speed. Then, we compared the fire-FRD-CNN with other models on the KITTI leader board. We used a single NVIDIA 1080 Ti GPU for our experiments. Code was implemented in TensorFlow and compiled with cuDNN.

In the experiments, we used input images in the KITTI dataset with their original resolution. Because the test dataset in KITTI has no ground truth information, we split the trainval dataset into training and validation datasets. In all our experiments, the training dataset was used for training, and the validation dataset was used for testing. For learning, a stochastic gradient descent with a momentum of 0.9 was used for optimization. We set the initial learning rate to 0.001. The learning rate was divided by 10 every 40,000 iterations. We used a batch size of 20. The number of default boxes was set to 9.

#### Average precision

We evaluated the detection accuracy of our proposed model on the KITTI dataset by average precision (AP). Average precision is defined based on precision and recall. In a classification task, the precision and recall for a class is defined as

6 $$\begin{array}{l}\Pr ecision=\frac{TP}{TP+FP},\end{array}$$

7 $$\begin{array}{l}Recall=\frac{TP}{TP+FN},\end{array}$$

where TP is the number of true positives, i.e., the number of items correctly labeled as belonging to the class; FP is the number of false positives, i.e., the number of items incorrectly labeled as belonging to the class; and FN is the number of false negatives, i.e., the number of items that were not labeled as belonging to the class but do in fact belong to that class.

We use intersection over union (IOU) to measure the location accuracy. The localization is more accurate if the IOU is larger. If the IOU of the predicted boxes and the ground truth is larger than 0.5, the predicted boxes are classified into a positive sample set. The neural network attempts to learn a set of transaction parameters to promote location accuracy. If the IOU of the predicted boxes and the ground truth is smaller than 0.5, the predicted boxes are classified into a negative sample set. In this case, the location loss is set to zero, and the neural network does not learn the transaction of the negative samples.

When evaluating an object detection framework using precision or recall, if we emphasize only high precision of a framework, the recall is often very low, and vice versa. Based on different thresholds of recall, one can plot a recall-precision curve, and a function of precision and recall can be constructed. To obtain a norm that reflects the whole performance of a framework, the average precision is introduced. The average precision has several forms in different areas, such as the area that is surround by the x-axis, y-axis, and the recall-precision curve. In our work, we follow the method proposed by the Pascal VOC 2007 dataset, which computes the average precision by averaging the precision over a set of evenly spaced recall levels 0, 0.1, 0.2, … 1.0:

8 $$\begin{array}{l}Average\,Precision=\frac{1}{11}\sum _{r\in (0,0.1,0.2,\ldots ,1.0)}\,{P}_{interp}(r),\end{array}$$

where *P*_*interp*_(*r*) is an interpolated precision that takes the maximum precision over all recalls greater than *r*.

We list the AP values of several models used autonomously in Table 3. As we can see in the table, the fire-FRD-CNN achieves better AP values in 8 categories compared to the original SqueezeDet+ model. The number of parameters of FRD-CNN is one times larger than SqueezeDet+, while also 9.5 times and 38 times smaller than RRC and MS-CNN, respectively. We propose that the performance improvement is mainly due to the innovative architecture.

**Table 3:** Summary of detection results of different models on KITTI object detection challenge.

Methods	car (e)	car (m)	car (h)	pede strian (e)	pede strian (m)	pede strian (h)	cyclist (e)	cyclist (m)	cyclist (h)	Number of parameters
RRC	—	89.85	—	—	75.33	—	—	76.47	—	148M
MS-CNN	90.03	89.02	76.11	83.92	73.70	68.31	84.06	75.46	66.07	551M
SqueezeDet	90.2	84.7	73.9	77.1	68.3	65.8	82.9	75.4	72.1	2M
SqueezeDet+	90.4	87.1	78.9	81.4	71.3	68.5	87.6	80.3	78.1	6.7M
fire-FRD-CNN (ours)	93.1	88.4	79.7	84.2	76.8	69.2	87.9	83.6	79.6	14M

#### Recall

For some applications, such as autonomously driven vehicles, recall is essential for safety; therefore, we analyze the recall of our proposed fire-FRD-CNN model. The fire-FRD-CNN generates 15,048 bounding box predictions for each image using their original resolutions. It is difficult to perform nonmaximum suppression (NMS) directly on so many bounding boxes because too many bounding boxes results in more time being spent on inference tasks. Therefore, we performed NMS only on bounding boxes with the top 64 confidence values and excluded other bounding boxes. Because there exists a trade-off between time spent on inference and recall tasks, the number 64 was selected based on cross validation. Finally, the recall value is 83.6%.

#### Speed

Inference speed is essential for some applications, such as in autonomously driven vehicles where fast reactions to the environment are required. If the inference speed does not satisfy real-time demand, our model cannot be used in a real environment. Our proposed fire-FRD-CNN model achieves real-time inference speed on the KITTI dataset. The inference speed is 0.029 seconds per image and 34.5 frames per second.

### Experiments on PASCAL VOC 2007, PASCAL VOC 2012, and COCO datasets

The PASCAL VOC 2007 and 2012 datasets^4^ contain 20 categories and over 11,000 images and 27,000 targets. They provide standardized image datasets for object detection, image segmentation, and other computer vision tasks. COCO is a large dataset^22^ that solves three core research problems in scene understanding: detecting noniconic views of objects, contextual reasoning between objects, and the precise 2D localization of objects. To further evaluate the proposed model, we performed two sets of control experiments on the PASCAL VOC and COCO datasets. The first set of experiments was combined with PASCAL VOC 2007 trainval and PASCAL VOC 2012 trainval sets to train the model; then, we evaluated the model on the PASCAL VOC 2007 test set. The second set of experiments was combined with the PASCAL VOC 2007 trainval, PASCAL VOC 2012 trainval, and COCO datasets for training, and the model was evaluated on the PASCAL VOC 2007 test set. These experimental settings were adopted in many experiments to evaluate the proposed framework.

When training the model, we selected the size of the batch as 20 and the size of the input image as 300 × 300. For training stability, the initial learning rate was set to 0.0001; to make the training jump out of the local minimum, the learning rate was increased to 0.005 in 500 rounds. As the training gradually converged, it decreased to 0.0001 at 80,000 rounds; it then decreased to 0.00001 at 100,000 rounds. The training ended at 120,000 rounds. A larger batch size is a good choice for batch normalization. However, the larger the batch size, the more memory consumed, especially when input images are relatively large. For the KITTI dataset, we used original images without the downsampling operation for better detection results. We set the batch size to 14 for a better trade-off.

The first set of experiments used PASCAL VOC 2007 trainval and PASCAL VOC 2012 trainval to train the model, and then we evaluated the model on the PASCAL VOC 2007 test set. We used the mAP value to evaluate the model, and the results are shown in Table 4. The proposed model had a mAP value of 77.95 on the VOC dataset. Additionally, the detection speed was 37 frames per second, which allows for real-time detection.

**Table 4:** Evaluation results of the fire-FRD-CNN on the VOC 2007 test set.

class	chair	motorbike	bottle	cat	potted plant
AP	59.97	83.71	50.31	87.25	53.78
**class**	**train**	**car**	**person**	**cow**	**sheep**
AP	86.41	85.20	80.13	85.23	80.96
**class**	**dog**	**bicycle**	**boat**	**dining table**	**horse**
AP	85.73	83.11	73.81	77.42	86.46
**class**	**bird**	**airplane**	**sofa**	**TV monitor**	**bus**
AP	76.24	82.33	79.26	75.77	85.99

Table 5 shows that the proposed model has a mAP value of 77.95 on the VOC 2007 test set. We present the evaluation results of the proposed and existing models in Table 5.

**Table 5:** Comparison of evaluation results between fire-FRD-CNN and other models on the VOC test set.

Model	Train	Test	mAP	FPS
SSD500^21^	VOC 2007 + 2012	VOC 2007	76.8	19
YOLOv2^23^	VOC 2007 + 2012	VOC 2007	76.8	67
SSD300^21^	VOC 2007 + 2012	VOC 2007	74.3	46
YOLOv1^24^	VOC 2007 + 2012	VOC 2007	63.4	45
SSD + Global-Local^9^	VOC 2007 + 2012	VOC 2007	79.6	39.5
SSD + Global-Local + CL^10^	VOC 2007 + 2012	VOC 2007	79.5	40.5
Efficient FPN^11^	VOC 2007 + 2012	VOC 2007	80.4	111
StairNet^12^	VOC 2007 + 2012	VOC 2007	78.8	30
DiSSD300^13^	VOC 2007 + 2012	VOC 2007	78.1	40.8
DeSSD300^13^	VOC 2007 + 2012	VOC 2007	77.6	39.8
FRD-CNN (ours)	VOC 2007 + 2012	VOC 2007	77.95	37

The second set of experiments used the PASCAL VOC 2007 trainval, PASCAL VOC 2012 trainval, and COCO datasets for training. The model was evaluated on the PASCAL VOC 2007 test set. We used the mAP values to evaluate the model and present the results in Table 6.

**Table 6:** Evaluation results of the fire-FRD-CNN on the VOC 2007 test set.

class	airplane	bicycle	bird	boat	bottle
AP	91.9	88.7	84.9	75.8	71.2
**class**	**bus**	**car**	**cat**	**chair**	**cow**
AP	86.5	87.4	94.6	66.5	89.7
**class**	**dining table**	**dog**	**horse**	**motor bike**	**person**
AP	69.4	93.7	91.6	90.9	89.9
**class**	**potted plant**	**sheep**	**sofa**	**train**	**TV monitor**
AP	67.5	88.6	76.7	90.4	80.0

Table 7 shows that the proposed model has a mAP value of 83.8 on the VOC 2007 test set. We present the evaluation results of the proposed and existing models in Table 7.

**Table 7:** Comparison of evaluation results between the fire-FRD-CNN and other models on the VOC test set.

Model	Train	Test	mAP	FPS
SSD500^21^	VOC 2007 + 2012 + COCO	VOC 2007	82.2	19
YOLOv2^23^	VOC 2007 + 2012 + COCO	VOC 2007	81.5	67
SSD300^21^	VOC 2007 + 2012 + COCO	VOC 2007	79.3	46
YOLOv1^24^	VOC 2007 + 2012 + COCO	VOC 2007	57.9	45
FRD-CNN(ours)	VOC 2007 + 2012 + COCO	VOC 2007	83.8	37

### Experiments on mobile units

We applied the proposed feature reuse methods on mobile units. We call it mobile-FR-CNN. We used mobile-FR-CNN as the backbone network and evaluated it on the VOC dataset. Mobile-FR-CNN parameters are listed in Table 1, where * is a parameter that numerically is the same as the total number of channels of feature maps after concatenation operation. We used the MultiBox detector proposed in SSD.

When training the model, we selected the size of the batch as 24 and the size of the input image as 300 × 300. To make the training jump out of the local minimum, we used an initial learning rate of 0.01 at the first 40 k iterations, then continued training with a learning rate of 0.001 until 80 k iterations, and the learning rate decreased to 0.0001 and 0.00001 at 100 k and 120 k, respectively. The training stopped when the model fully converged at 140 k iterations. We used a pretrained MobileNet to initialize the layers of mobile FR-CNN before the first concatenation operation. The layers after the first concatenation operation layer were initialized by random values.

The experiments used PASCAL VOC 2007 trainval and PASCAL VOC 2012 trainval to train the model, and then we evaluated the model on the PASCAL VOC 2007 test set. We used the mAP value to evaluate the model, and the results are shown in Table 8.

**Table 8:** Comparison of evaluation results between the mobile-FRD-CNN and the MobileNet.

Model	Train	Test	mAP	Number of parameters
MobileNet + SSD detector	VOC 2007 + 2012 + COCO	VOC 2007	72.7	6.8M
Mobile-FRD-CNN (ours)	VOC 2007 + 2012 + COCO	VOC 2007	74.9	8.4M

From Table 8, we can see that Mobile-FRD-CNN had a mAP value of 74.9 on the VOC dataset. The mAP value of it had a 2.2 percentage improvement compared to MobileNet with SSD detector. The number of parameters of it was 0.2 times larger than MobileNet with SSD detector. Mobile-FRD-CNN achieved better performances with small increases in the number of parameters. We believe that the performance improvement was mainly due to the innovative architecture.

## Discussion

In this paper, we proposed a method that fuses the good features of fire units, mobile units and different feature reuse methods. First, we constructed the fire-FR-CNN and mobile-FR-CNN. Then, we applied bypass and complete concatenation paths on different layers of the CNN. Finally, we integrated an object detection network to construct the fire-FRD-CNN and mobile-FRD-CNN. Experiments showed that the proposed methods had relatively stable improvements compared to the original SqueezeDet and MobileNet models. Through analysis of the experiment, we concluded that this is because the two types of feature reuse methods—skip connection and dense connection—fuse feature information from layers of different scales, which is very useful for object detection. In addition, the skip connection and dense connection also overcome the vanishing gradient problem. Fire-FRD-CNN and mobile-FRD-CNN models are also relatively small in size. They can be applied to autonomously driven and mobile devices where storage is critical. As a result, the limitations of dense connectivity, which incur very large model sizes in many applications, were addressed.

Fire units and mobile units compress model size based on different mechanisms. Fire units compress model size through squeeze layers; mobile units compress model size through depthwise separable convolutions. Because SqueezeDet and MobileNet have already been tested on the KITTI dataset and mobile devices, respectively, and our experiments were based on their works, we suggested that fire-FRD-CNN be used in autonomously driven vehicles and mobile-FRD-CNN be applied to mobile devices in the current stage. In the future, we will apply the proposed backbone models to other datasets and computer vision tasks, such as image segmentation, to demonstrate their versatility.
